# By-Products of Camu-Camu [*Myrciaria dubia* (Kunth) McVaugh] as Promising Sources of Bioactive High Added-Value Food Ingredients: Functionalization of Yogurts

**DOI:** 10.3390/molecules25010070

**Published:** 2019-12-24

**Authors:** Natália Conceição, Bianca R. Albuquerque, Carla Pereira, Rúbia C. G. Corrêa, Camila B. Lopes, Ricardo C. Calhelha, Maria José Alves, Lillian Barros, Isabel C. F. R. Ferreira

**Affiliations:** 1 Centro de Investigação de Montanha (CIMO); Instituto Politécnico de Bragança, Campus Santa Apolónia, 5300-253 Bragança, Portugal; natalia.conceicao@ifro.edu.br (N.C.); bianca.albuquerque@ipb.pt (B.R.A.); carlap@ipb.pt (C.P.); rubiacorrea@ipb.pt (R.C.G.C.); calhelha@ipb.pt (R.C.C.); maria.alves@ipb.pt (M.J.A.); 2 Instituto Federal do Rondônia—IFRO, Campus Colorado do Oeste, Rondônia 76993-000, Brazil; camila.lopes@ifro.edu.br; 3 Program of Master in Science, Technology and Food Safety, Cesumar Institute of Science Technology and Innovation (ICETI), University Center of Maringá (UniCesumar), Maringá, Paraná 87050-390, Brazil

**Keywords:** *Myrciaria dubia*, hydroethanolic extracts, phenolic composition, antibacterial potential, anti-proliferative effects

## Abstract

Camu-camu (*Myrciaria dubia* (Kunth) McVaugh) is a fruit economically relevant to the Amazon region, mostly consumed in the form of processed pulp. Our aim was to perform an unprecedented comparative study on the chemical composition and bioactivities of the camu-camu pulp and industrial bio-residues (peel and seed), and then the most promising fruit part was further explored as a functionalized ingredient in yogurt. A total of twenty-three phenolic compounds were identified, with myricetin-*O*-pentoside and cyanindin-3-*O*-glucoside being the main compounds in peels, followed by *p*-coumaroyl hexoside in the pulp, and ellagic acid in the seeds. The peel displayed the richest phenolic profile among samples, as well as the most significant antibacterial (MICs = 0.625–10 mg/mL) and anti-proliferative (GI_50_ = 180 µg/mL against HeLa cells) activities. For this reason, it was selected to be introduced in a food system (yogurt). Taken together, our results suggest the possibility of using the camu-camu peel as a source of food additives.

## 1. Introduction

Camu-camu (*Myrciaria dubia* (Kunth) McVaugh), also known as caçari and araçá d’água, is a tropical fruit belonging to the Myrtaceae family, often found in flooded regions of the Amazon rainforest [1]. Its round berries are about 2.5 cm in diameter, containing one to four seeds. The fruit possess a red-to-purple bright pericarp and a very tart acid, juicy, and pink mesocarp [2]. A member of the so-called group of super fruits, camu-camu is known by its superlative ascorbic acid content (up to 2.780 mg per 100 g of fresh fruit) and by its content of other bioactive molecules such as anthocyanins (cyanidin-3-*O*-glucoside and delphinidin-3-*O*-glucoside), flavonols (myricetin, quercetin), ellagic acid, ellagitannins, proanthocyanidins, and carotenoids (lutein, β-carotene, violaxanthin and luteoxanthin) [1,3,4]. These phytochemicals display neutralizing properties of reactive species related to anti-obesogenic, hypolipidemic, anti-inflammatory, anti-genotoxic, and neuroprotective outcomes have been verified in in vitro and in vivo experiments, as well as clinical trials [4,5,6,7,8]. 

*Myrciaria dubia* is economically relevant to the Amazon region, as it grows in low-value areas that are usually inadequate for crops of other species [5]. Due to its very high acidity, the camu-camu fruit is not consumed in natura. Instead, it is used in the form of juices, purees, and mostly pulp, applied in beverage production and as a food ingredient [5,9]. The industrial processing of *M. dubia* pulp generates by-products, which create an environmental problem. Considering that up to 40% of the fruit mass is composed of seeds and peels, in the past years, studies have been carried out, aiming to reduce the environmental impact and economically exploit these abundant by-products. There has been previous work on the biological activities of camu-camu by-products [10], including its anticancer potential [5], as well as on strategies for the recovery of polyphenols from these materials [11].

Natural extracts with antioxidant properties can be employed as substitutes for artificial additives and also display a potential role in the prevention of diseases related to oxidative stress [12]. In the same way, the antimicrobial properties of some active phytochemicals could delay or inhibit the growth of pathogenic and/or toxin-producing microorganisms in food, thus avoiding both foodborne diseases and food spoilage [13]. Therefore, natural extracts can combine the functions of preservative and functionalizing additive, while simultaneously improving the food stability and nutritional value [14].

Following the sustainability principles implemented in today’s society, new high-added-value compounds are being recovered from agri-food by-products and applied in the development of food ingredients with preserving capacity, thereby promoting resource-use efficiency and circularity [15]. Despite the functional attributes verified for the *M. dubia* by-products [9,11,16,17], their exploitation as food additives is still very limited [11].

The successful application of extracts of plants, mushrooms, and their bio-residues as preservative and/or fortifying ingredients in yogurt formulation has been largely demonstrated by our group [12,18,19]. To the best of our knowledge, there are only two studies on the application of camu-camu extracts in food production [11,20]. Given the above, the aim of this study was to perform an unprecedented comparative investigation on the biological activities of *Myrciaria dubia* extracts obtained from its fruit pulp and by-products (i.e., peel and seeds). The antimicrobial, anti-proliferative, and hepatotoxic potentials of the extracts were evaluated, and the most active one was tested as a functionalizing ingredient in yogurt. Furthermore, the nutritional composition, pH, and fatty acids composition of the enriched dairy product were investigated, along with shelf life (0, 7, and 15 days).

## 2. Results and Discussion 

### 2.1. Phenolic Compounds Profiles

The chromatographic and mass spectrometric data of the phenolic compounds from the hydroethanol extracts of different parts of *M. dubia* fruits are displayed in Table 1. A total of twenty-three compounds were identified, comprising twelve phenolic acids (nine ellagic acid derivatives, caffeoylquinic acid, p-coumaric acid, and ferulic acid derivatives), three flavonols (myricetin derivatives), one flavone (apigenin derivative), three chalcone/flavanone derivative ((iso)liquiritigenin glycoside derivatives), two anthocyanins, and two hydrolysable tannins. As shown in Table 1, peak 1 ([M − H]^−^ at *m*/*z* 353) was tentatively identified as 4-*O*-caffeoylquinic acid based on the fragmentation pattern previously described [21], being identified for the first time in camu-camu fruit. Peak 2 ([M − H]^−^ at *m*/*z* 325) presented a unique MS^2^ fragment at *m*/*z* 163 and according with previous studies in this fruit [3], the fragmentation suggested the presence of a *p*-coumaric acid with loss of an hexoside moiety (162 u), being tentatively identified as *p*-coumaroyl hexoside. This compound was the main phenolic acid derivative found in the *M. dubia* pulp.

Peaks 3, 4, and 5 presented the same pseudomolecular ion ([M − H]^−^ at *m/z* 417) and a unique MS^2^ fragment at *m/z* 255 ((iso)liquiritigenin aglycone), revealing the loss of hexoside moiety (162 u), thus being tentatively assigned as (iso)liquiritigenin-*O*-hexoside. The fragment at *m/z* 255 can be characterized as a liquiritigenin (a flavanone) or isoliquiritigenin (a chalcone). As the fragmentation patterns do not allow to distinguish between both compounds, they were assigned as derived from one or another based on their UV spectra [22]. This phytochemical, commonly found in *Glycyrrhiza* ssp., was detected for the first time in *M. dubia* extracts.

Peaks 6 ([M − H]^−^ at *m/z* 449), 7 ([M − H]^−^ at *m/z* 463), and 16 ([M − H]*^−^* at *m/z* 479) presented a unique MS^2^ fragment at *m/z* 317 corresponding to myricetin glycoside derivatives, bearing a pentosyl (−132 u), deoxyhesoxosyl (−146 u) and hexosyl (−162 u) moieties, respectively. All three compounds have been previously identified in camu-camu fruit [1,3]. They were designated as myricetin-*O*-pentoside, myricetin-*O*-deoxyhexoside, and myricetin-*O*-hexoside, and they were the major components found in the peel extract. Similarly, peak 8 ([M − H]^−^ at *m*/*z* 431) revealed a unique MS^2^ fragment at *m*/*z* 269 (apigenin aglycone) being tentatively assigned as apigenin-*O*-hexoside, as previously described [3].

Ellagic acid derivatives (peaks 9–12 and 19–23) were the main molecules detected in the *M. dubia* seeds. The principal phenolic of the seed extract, ellagic acid (peak 21), was identified, taking into account the MS fragmentation pattern as also the UV-Vis in comparison with the commercial standard. Peaks 9 and 10 ([M − H]^−^ at *m*/*z* 719) were identified in camu-camu fruits [1]. A complete characterization of this compound has not yet been fully elucidated; consequently, these peaks were tentatively identified as ellagic acid derivatives. Similarly, peaks 11 and 12 ([M − H]^−^ at *m/z* 733) were also identified as ellagic acid derivatives. Peaks 19 ([M − H]^−^ at *m/z* 463), 20 ([M − H]^−^ at *m/z* 433), and 22 and 23 ([M − H]^−^ at *m/z* 489) were identified as ellagic acid derivatives, bearing a hexosyl (162 u), pentosyl (132 u), and acetyl rhamnoside (–146 u), which were tentatively identified as ellagic acid hexoside, ellagic acid pentoside, and ellagic acid acetyl rhamnoside, respectively. Fracassetti et al. [1] have reported the occurrence of these molecules in camu-camu fruit.

Peak 15 ([M − H]^−^ at *m/z* 355) revealed a unique MS^2^ fragment at *m/z* 193 (ferulic acid), being assigned as ferulic acid hexoside. Ferulic acid has been previously identified in *M. dubia* [3]; thus, our study revealed the presence of the glycoside form of this phenolic acid. Peak 17 ([M − H]^−^ at *m/z* 469) was identified as valoneic acid dilactone, taking into account the fragmentation pattern described earlier in fruits camu-camu [1]. Peak 18 ([M − H]^−^ at *m/z* 935) presented fragmentation pattern characteristics of Di-HHDP-galloyl-glucoside, as also previously identified [1].

Peaks 13 ([M + H]^+^ at *m/z* 465) and 14 ([M + H]^+^ at *m/z* 449) identified in comparison with the chromatographic and MS fragmentation pattern in relation to the commercial standards, were recognized as delphinidin-3-*O*-glucoside (D3G) and cyanindin-3-*O*-glucoside (C3G), respectively. Both compounds were found in the peels, whereas the pulp only presented C3G, this molecule being the most abundant anthocyanin in either fruit parts. Zanatta et al. [23] also reported this anthocyanin profile for the whole fruit of fresh camu-camu.

In the past decade, the phenolic composition of the camu-camu fruit pulp has been studied by several authors, using chromatographic approaches, such as LC–TOF-MS [4] and UHPLC–MS/MS [3]. In addition to Vitamin C, the presence of other bioactive phytochemicals has been reported: anthocyanins, such as C3G and D3G; flavonols, mainly myricetin and quercetin; ellagic acid; ellagitannins; proanthocyanidins; and also carotenoids, like lutein, β-carotene, violaxanthin, and luteoxanthin [1,3,4,24]. 

The peel extract presented the richer phenolic profile (9 and 2, non- and anthocyanin compounds, respectively), which was predominantly composed of myricetin-*O*-pentoside, apigenin-*O*-hexoside, and C3G, in addition to the highest total phenolic content (TPC) (33.4 ± 0.5 mg/g). This was followed by the seed extract, which had eight different phenolic constituents identified, being the most plentiful ones ellagic acid and an ellagic acid pentoside, and a slightly lower TPC value (23.41 ± 0.07 mg/g). Regarding the pulp extract, only five phytocomponents were detected, the principal being a p-coumaric acid hexoside, and only one anthocyanin compound (C3G); furthermore, this sample displayed a much lower TPC value in comparison to the other camu-camu fruit parts (4.32 ± 0.03 mg/g). Fracassetti et al. [1], when analyzing mature fruits from Peru, found a close TPC value in the camu-camu seeds (3.36 mg/g), which was interestingly higher than the one verified for its corresponding pulp sample (0.086 mg/g). Gonçalves et al. [24] reported a TPC of almost 25 mg CAE/100 g dry weight (DW) for a camu-camu frozen pulp sample.

Regarding anthocyanin compounds, C3G was the major one detected in the peel (4.68 ± 0.07 mg/g DW) and the unique in the pulp (1.000 ± 0.001 mg/g DW). In contrast with our study, Zanatta et al. [23] found five anthocyanin compounds (D3G, C3G, one cyanidin derivate, and two nonidentified anthocyanins) in whole camu-camu, and Fracassetti et al. [1] detected only C3G in the pulp and peel. This fact can be justified not only by the different edaphoclimatic conditions but also, and mainly, to the maturation stage of the assessed fruits. Neves et al. [25] monitored the levels of bioactive phytochemicals in camu-camu fruits throughout development. They confirmed that the flavonoid contents increase during maturation. They also verified that the highest pigment and vitamin C concentrations occurred in the fruit peels. The presence of anthocyanin compounds was not detected in the camu-camu seeds, as already reported by Fracassetti et al. [1].

The phenolic composition of the camu-camu fruit residues has also been previously determined by Fracassetti et al. [1], who studied the phenolic composition of two powders obtained from mature camu-camu fruits: a dehydrated pulp powder and a dried flour composed of peel and seeds (residues from pulp extraction). More than fifty phytocomponents were detected in these samples via HPLC–DAD–ESI-MS–MS and UPLC–HR-QTOF–MS–MS. Among these, myricetin, ellagic acid, and ellagitannins were present in both samples; quercetin was detected exclusively in the pulp powder, whereas proanthocyanidins only in the residue flour. Later, Kaneshima et al. [16] isolated six tannins with great antioxidant capacity from camu-camu seed and peel acetone extracts, namely casuarinin, castalagin, grandinin, methylvescalagin, stachyurin, and vescalagin. However, to our best knowledge, the present work is the first comparative in-depth study on the phenolic profiles of the distinct components of the camu-camu industrial residue, i.e., peel and seeds.

### 2.2. Evaluation of Bioactive Properties

The in vitro antimicrobial, anti-proliferative, and hepatotoxic potentials of the camu-camu ethanolic extracts were evaluated, and the results are presented in Table 2 and Table 3. The antimicrobial potential of the three camu-camu extracts was evaluated against five Gram-negative bacteria (*Escherichia coli, Klebsiella pneumoniae, Morganella morganii, Proteus mirabilis,* and *Pseudomonas aeruginosa*), four Gram-positive bacteria (*Enterococcus faecalis, Listeria monocytogenes,* MRSA— Methicillin-resistant *Staphylococcus aureus,* and MSSA—Methicillin-susceptible *Staphylococcus aureus*) and one fungal strain (*Candida albicans*). The extracts’ minimum inhibitory concentrations (MIC), minimum bactericidal (MBC), and minimum fungicidal (MFC) concentration are displayed in Table 2. Overall, the camu-camu extracts were more active against Gram-positive bacteria than against Gram-negative bacteria. Kaneshima et al. [17] observed the same tendency for n-hexane extracts from camu-camu peel and seeds. 

Our *M. dubia* peel extract displayed the most relevant antimicrobial potential among samples, followed by the pulp and the seed extracts, with MIC values ranging between 0.625 (great inhibitory activity against MRSA and MSSA) and 10 mg/mL (for *E. coli*, *K. pneumoniae, P. mirabilis,* and *E. faecalis*). Carranza and Nelly [26] found less notorious results in their study on the inhibitory effects of camu-camu ethanolic peel extract against *S. aureus* and *C. albicans*: MIC values of 750 and 1000 mg/mL, respectively. Moyda et al. [27] reported that the methanolic extract of camu-camu juice residue (seeds and peel) was active against *S. aureus*, and attributed this bioactivity to the extract’s lipophilic components. Kaneshima et al. [17] confirmed that acylphloroglucinol and rhodomyrtone are antimicrobial constituents of the *M. dubia* peel. 

Regarding our pulp extract, it was significantly active against *M. morganni* (MIC = 5 mg/mL). Camere-Colorassi et al. [6] evaluated the in vitro antibacterial activity of methanolic extracts of camu-camu pulp and seeds against *Streptococcus mutans* and *Streptococcus sanguinis* by the agar diffusion and microdilution methods. Their methanolic pulp extract had an effect of 16.20 mm and 19.34 mm against *S. mutans* and *S. sanguinis*, respectively, whereas a MIC value of 62.5 mg/mL for both strains. Our camu-camu seed extract had the most relevant antimicrobial activity against *L. monocytogenes* (MIC of 2.5 mg/mL) and the fungal strain *C. albicans* (MIC of 10 mg/mL) among tested samples. Camere-Colorassi et al. [6] reported a significant antibacterial effect for the methanolic seed extract of camu-camu against *S. mutans* and *S. sanguinis*, with inhibitory halos of 21.36 and 19.21 mm, respectively.

The antimicrobial responses observed in our study likely come from the multiple mechanisms and synergistic effects of various bioactive phytocomponents of the camu-camu extracts [28]. We believe that the promising antibacterial potential of the peel extract may relate, inter alia, to its composition in derivatives of ellagic acid and myricetin (Table 1). Gomes et al. [29] found that a hydromethanolic extract of *Eucalyptus globulus* Labill. rich in ellagic acid derivatives has great antibacterial effectiveness against *S. aureus*, the dairy industry pathogen. Furthermore, Arita-Morioka et al. [30] reported that myricetin derivatives display inhibitory effects in biofilm formation by wild-type *E. coli* K12 in liquid culture.

Our extracts displayed MIC values above 1.6 mg/mL for almost all the microorganisms tested (Table 2), results that, in accordance to some authors, are features of weak inhibitors [28]. One should consider that the bacteria strains applied in our tests are clinical isolates of multi-resistant strains, which present significantly higher antibiotic resistance profiles than bacterial strains from American Type Culture Collection (ATCC). Accordingly, our data can be interpreted as being indicative of promising antibacterial potential.

Table 3 shows the anti-proliferative activity of the camu-camu ethanolic extracts toward four human tumor cell lines, expressed as the concentrations that allow 50% of cell growth inhibition (GI_50_). The peel extract was the only sample that displayed inhibitory effects against all tested lines, besides the greatest capacity values, being the most expressive result against cervical carcinoma (HeLa) cells (GI_50_ = 180 ± 8 µg/mL). However, the pulp extract also presented significant cytotoxic activity (GI_50_ = 196 ± 7 µg/mL against HeLa line), followed by the seed extract. Herrera-Calderon et al. [7] also investigated the cytotoxic potential of *M. dubia* ethanolic extract against various human tumor cell lines, among which H-460 (lung large cell carcinoma) and MCF-7 (breast carcinoma), obtaining GI_50_ values of 26.78 and 35.44 µg/mL, respectively. The suggestive anti-proliferative activity for our *M. dubia* peel extract possibly relate to its content in derivatives of apigenin and myricetin (Table 1). The apigenin derivatives isolated from the hydroethanolic extract of *Selaginella doederleinii* Hieron. displayed substantial in vitro cytotoxic effects against lung cancer cells (A549 line), with GI_50_ values ranging from 0.82–1.32 µg/mL [31]. Likewise, a C-glycosylated derivative of apigenin isolated from the hydroethanolic leaf extract of *Ocimum basilicum* var. thyrsiflorum (L.) Benth. (large leaf basil) exhibited significant cytotoxicity against the HCT116 human colon cancer cell line [32]. Further, in the past years, several in vitro and in vivo studies have demonstrated the anti-proliferative and chemotherapeutic actions of myricetin against several cancer types [33].

All the studied *M. dubia* extracts have shown no toxicity against the liver primary culture PLP2, seeing that their GI_50_ values were higher than the highest concentration assessed (400 μg/mL) (Table 3). Camere-Colarossi et al. [6] assessed the toxicity of seed and pulp methanolic extracts of *M. dubia* in canine kidney cells (MDCK cell line), using the succinate dehydrogenase activity assay (MTT method) and found IC_50_ values of 725 and 424.37 μg/mL, respectively. Both samples displayed no cytotoxicity against the MDCK line. Akachi et al. [34] verified that a 1-methylmalate isolated from *M. dubia* had suppressive effects on GalN-induced liver injury in rats, which suggests a potential hepatoprotective activity of the camu-camu fruit juice.

The application of plant extracts as additives and/or nutraceuticals mandatorily demands proof of its toxicological features [28]. Indeed, the absence of hepatotoxicity herein confirmed for the camu-camu extracts not only corroborates the above-cited studies, but also endorses their safe use in food systems.

### 2.3. Fortification of Yogurt with Camu-Camu Peel Extract

To verify the feasibility of using the bioactive peel extract as a natural food preservative and fortifier, the characterization of the enriched dairy product, which included the determination of the nutritional and fatty acids profiles, was performed. Both control (IC) and fortified (IF) samples were analyzed at three time periods: immediately after incorporation, after seven days of storage at 4 °C, and after 15 days of storage at 4 °C. The obtained results are displayed in Table 4 and Table 5. 

In general, the addition of camu-camu peel extract to the yogurt did not significantly change the product’s nutritional profile. As expected, and despite the adequate storing conditions, there was a very slight moisture loss over the period (without statistical significance), which was reflected in a minimal increase in samples’ energy value. The average moisture content (83.4 ± 0.9 g/100 g) verified for IF is slightly lower than the average values found by Caleja et al. [12] (87.5 g/100 g) for homemade yogurts enriched with *Matricaria recutita* L. (chamomile) and *Foeniculum vulgare* Mill. (fennel) decoctions, and by Oliveira et al. [19] (88.5 g/100 g) for their yogurts functionalized with a hydroethanolic flower extract of *Arenaria montana* L. IF’s total carbohydrate content (average of 5.4 ± 0.3 g/100 g) was superior to the value found for Oliveira et al. [19] (4 g/100 g). However, it was identical to the content verified by Corrêa et al. [14] (5.4 g /100 g) for yogurt enriched with ergosterol from *Agaricus blazei* Murril. bioresidues. Our control and fortified samples presented high protein contents, with average values of 6.4 ± 0.5 and 6.3 ± 0.3 g/100 g, respectively, which were superior to those reported by other authors [14,19].

The free sugars galactose (average of 1.12 ± 0.07 g/100 g) and lactose (average 2.8 ± 0.2 g/100 g) were detected in our yogurt samples (Table 4). While the galactose content remained constant, the lactose content increased minimally during the storage period. Corrêa et al. [14] found a similar content of galactose (1.01 g/100 g), whereas a higher amount of lactose (4.17 g/100 g).

Aguiar and Souza [20] formulated yogurts, using camu-camu fruit pulp (applied in the concentrations of 10%, 13%, and 15%), whole UHT milk, dairy culture, and sugar. The developed dairy products presented microbiological stability over a 30-day storage period, with chemical and physical parameters within the legislation limits. Furthermore, the yogurt with 10% camu-camu pulp had satisfactory sensory acceptance.

Twenty-five fatty acids (FAs) were detected in our yogurt samples, and their amounts were expressed in relative percentage (Table 5). The most plentiful FA was palmitic acid (C16:0), followed by oleic (C18:1n9), myristic (C14:0), and stearic (C18:0) acids. Heleno et al. [17] detected a similar FA profile in yogurts functionalized with mycosterols from *Agaricus bisporus* (J.E.Lange) Imbach. The general FA proportion (SFA > MUFA > PUFA) verified in our samples was expected and is in agreement with previous studies of our group in which fortified yogurts were characterized [12,14,18,19]. Overall, the addition of the *M. dubia* peel extract did not modify the yogurts’ FA profile, which remained virtually unchanged throughout the 15-day monitoring, especially with regard to the contents of the most important FAs (Table 5). Corrêa et al. [14] reported the same tendency. Although it is known that camu-camu pulp contains stearic, linoleic, oleic, γ-linolenic, α-linolenic, tricosanoic, and eicosadienoic fatty acids [2], the FA composition of the fruit peel, to our best knowledge, has not been reported.

In terms of pH, the differences between the control (4.35, 4.27, and 4.21 for 0, 7, and 15 days of storage, respectively) and the fortified (4.56, 4.47, and 4.39 for 0, 7, and 15 days of storage, respectively) samples were not statistically significant. The same observation could be done for each formulation over time.

In what concerns the phenolic compounds concentration in the yogurts, a decrease of about 25% and 30% was noticed after 7 and 15 days of storage, respectively. Nevertheless, once the incorporated dose was five times higher than the one required to provide bioactive properties to the yogurts (as described in Section 3.5), these properties were considered as guaranteed, despite the observed loss.

## 3. Materials and Methods 

### 3.1. Preparation of the Camu-Camu Extracts

Camu-camu (*Myrciaria dubia*) fruits were collected in an ecological reserve in Ji-Paraná, a city located in the Amazon region of Brazil. The voucher specimen is deposited at the Herbarium of the Federal Institute of Rondônia (IFRO), Western Colorado Campus (CO), under the number 1096. The fresh fruits were taken to the Laboratory of Microbiology of the IFRO-CO, washed, and manually separated into pulp, peels, and seeds. Each fruit part was frozen and subsequently freeze-dried (LIOTOP L101, São Paulo, Brazil). The samples were sent to the Mountain Research Center (CIMO), located at the Polytechnic Institute of Bragança (IPB), Portugal. 

Each extract was obtained by stirring (25 °C at 150 rpm) the lyophilized sample (1 g, ~20 mesh) with ethanol/water (80:20, *v*/*v*) for 45 min. Then, the mixture was filtered, using Whatman n° 4 paper, and the residue was submitted to extraction once again. At the end, the obtained extracts were combined, and the solvent was eliminated by using a rotary evaporator (Büchi R-210, Flawil, Switzerland) and lyophilization. 

**Figure 1:**
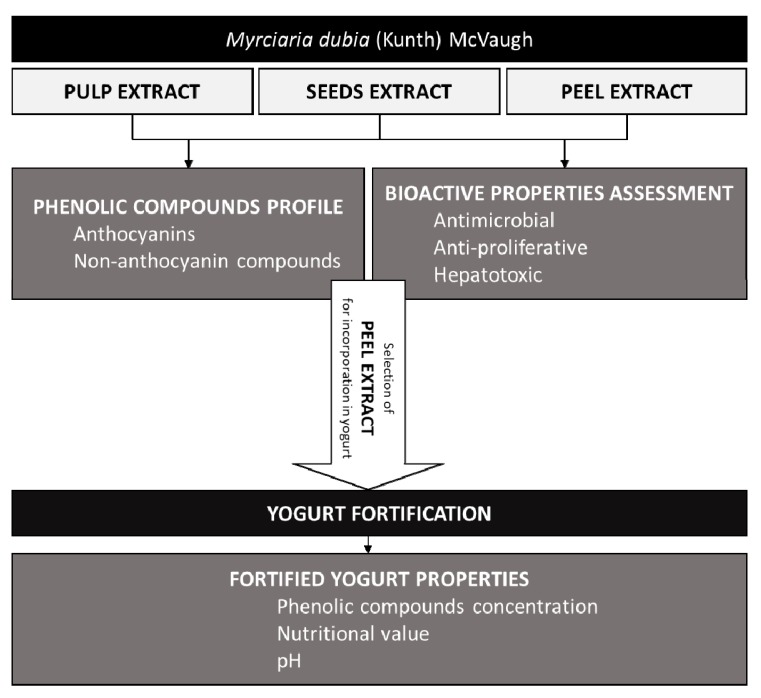
Overview of the experimental methodology.

For the anthocyanin analyses, the extracts were obtained by stirring the lyophilized sample (1 g) with 30 mL of ethanol/water (80:20 *v*/*v*) acidified with 0.1% citric acid (1 µM) for 60 min at room temperature and light protected. Afterward, the suspension was filtered (Whatman No. 4 filter paper), and the residue was re-extracted, using the same conditions. The combined filtrates were evaporated under reduced pressure (150 mbar) at 40 °C and subsequently lyophilized. An overview of the experimental methodology is presented in Figure 1.

### 3.2. Phenolic Compounds Analysis

For non-anthocyanin compounds, the freeze-dried camu-camu extracts were diluted in ethanol/water (80:20, *v*/*v*) at 5 mg/mL. The assessment of the samples phenolic profile was done by LC-DAD-ESI/MSn (Dionex Ultimate 3000 UPLC, Thermo Scientific, San Jose, CA, USA), following a methodology established by Bessada et al. [35]. Compound identification was accomplished by comparing their retention time, UV-vis, and mass spectra with those obtained from standard solutions, when available. Alternatively, peaks were tentatively identified, comparing the collected data with literature reports. Results were given in mg/g of lyophilized extract or mg/g of yogurt. 

For anthocyanins, lyophilized acidified ethanolic extracts obtained from different parts of camu-camu were dissolved (10 mg/mL) in acidified (0.1% of citric acid, 1 µM) ethanol/water (80:20 *v*/*v*) and filtered through a 0.22 µm disposable filter disk into a 1.5 mL amber vial for HPLC analysis. The anthocyanins profile was obtained by LC-DAD-ESI/MS (Dionex Ultimate 3000 UPLC, Thermo Scientific, San Jose, CA, USA), operating in positive mode following a methodology established by Gonçalves et al. [36]. Data were collected and analyzed, using the Xcalibur^®^ program (Thermo Finnigan). Results were presented in mg/g of lyophilized extract or mg/g of yogurt.

### 3.3. Antimicrobial Activity

The minimal inhibitory and bactericidal and fungicidal concentrations (MIC, MBC, and MFC) for all the bacterial strains and one yeast strain were determined, using the microdilution method, and the rapid *p*-iodonitrotetrazolium chloride (INT) colorimetric was used as microbial growth indicator as established by Pires et al. [37]. Five Gram-negative bacteria, four Gram-positive bacteria, and one yeast strain were used in this assay. The microorganisms used were clinical isolates donated from patients hospitalized in the Local Health of Bragança and Hospital Center of Trás-os-Montes and Alto-Douro Vila Real, Northeast of Portugal. The chosen positive controls were as follows: antibiotics ampicillin and imipenem for the Gram-negative bacteria; ampicillin and vancomycin for the Gram-positive; and fluconazole for the fungal strain.

### 3.4. Anti-Proliferative Activity and Hepatotoxicity

The anti-proliferative activity of the camu-camu ethanolic extracts was evaluated against four human tumor cell lines, namely MCF-7 (breast carcinoma), NCI-H460 (non-small cell lung cancer), HeLa (cervical carcinoma), and HepG2 (hepatocellular carcinoma), and its hepatotoxicity was assessed in a primary culture of porcine liver nontumor cells, PLP2 [38]. The sulforhodamine B (SRB) assay was performed according to the protocol established by the group and described in detail by Corrêa et al. [39]. Optical densities were read on an automated spectrophotometer plate reader at a single wavelength of 540 nm (Biotek Elx800). Results were expressed in GI_50_ values, which is the sample concentration required to inhibit 50% of the net cell growth; ellipticine was employed as positive control. 

### 3.5. Fortification of Yogurt with Camu-Camu Peel Extract

The camu-camu peel extract was the sample that presented the greatest amount of phenolic compounds, as well as the most relevant antimicrobial and anti-proliferative potentials. For this reason, it was the one chosen to be assessed into a food system (yogurt). Two groups of samples were prepared: (1) commercial yoghurt, without additives (control samples); and (2) yogurts enriched with camu-camu peel extract (fortified samples). The peel extract was incorporated into the yogurt in the concentration of 0.5%, in order to provide five times the highest MIC value verified in the antimicrobial assay. The natural plain organic yogurts were acquired at the local market of Bragança, Portugal. Samples were stored at 4 °C and evaluated over three different times (0, 7, and 15 days). Prior to analyses, samples were freeze-dried.

### 3.6. Nutritional Parameters

The proximate composition of the yogurts was evaluated, using the AOAC International standard methods [40]. Crude protein content (N × 6.38) was measured by the Kjeldahl assay; the crude fat was estimated by extracting a known weight of powdered sample with petroleum ether in a Soxhlet apparatus; the ash content was assessed by incineration at 550 ± 15 °C. Total carbohydrates were determined by difference, and the total energy by Equation (1):

Energy (kcal) = 4 × (g proteins + g carbohydrates) + 9 × (g lipids)(1)

Free sugars analysis was performed via HPLC coupled to refraction index detector. Quantification (g/100 g of yogurt) was achieved by using melezitose as the internal standard and identification by comparison with commercial standards. Fatty acids were assessed by GC coupled to an FID detector. Identification was possible by comparison with commercial standards, with results given in relative percentage of each fatty acid.

### 3.7. pH and Phenolic Compounds Evaluation

The pH of the yogurts (control and fortified samples) was measured with a HI 99161 pH-meter (Hanna Instruments, Woonsocket, Rhode Island, USA) by inserting the tip of the measuring pen into the yogurts. The measurement was performed in triplicate for each sample.

The phenolic composition of the yogurts was also assessed, as described in Section 3.2, in order to confirm their presence in the fortified samples. 

### 3.8. Statistical Analysis

Results were expressed as mean values and standard deviations (SD), based on three repetitions of the samples and respective concentrations. To ascertain the significant differences with α = 0.05 between less than three samples, a Student’s *t*-test was used. Analyses were performed with the IBM SPSS Statistics for Windows, version 23.0. (IBM Corp., Armonk, NY, USA).

## 4. Conclusions

To the best of our knowledge, this is the first comparative investigation on the in vitro antimicrobial and anti-proliferative capacities of the distinct parts of the camu-camu fruit, assessing separately its peel, pulp, and seeds. Our results show the potential bioactive of all fractions of this fruit, including its by-residues. Interestingly, camu-camu fruit by-products revealed a richer phenolic composition than the correspondent pulp, which increases the interest for exploration of whole fruit. Furthermore, the addition of *M. dubia* peel in a food matrix (yogurt) resulted in an enrichment in bioactive molecules in product without significantly altering its nutritional composition and fatty acid profile, suggesting that it may be used as ingredient to functional foods and/or food additive. Therefore, our results not only corroborate the relevance of the *Myrciaria dubia* fruit consumption to improve nutrition and health, but also indicate the possibility of using its industrial by-products as sources of valuable bioactive molecules, within the circular bioeconomy concept.

## Figures and Tables

**Table 1:** Retention time (Rt), wavelengths of maximum absorption in the visible region (λ_max_), mass spectrometric data, and tentative identification of phenolic compounds of the hydroethanolic extracts of the distinct parts camu-camu fruit investigated in the present work, namely peel, pulp, and seed.

Peak	Rt (min)	λ_max_ (nm)	[M − H]^−^/[M]^+^ *m*/*z*	Main MS^2^ Fragments (*m*/z)	Tentative Identification	Reference	Concentration (mg/g Extract)
**Peel**							
Non-anthocyanins							
1	6.0	324	353	191(22),179(45),173(100),161(10),135(5)	4-*O*-Caffeoylquinic acid	[21]	1.41 ± 0.04
2	7.2	314	325	163(100)	*p*-Coumaroyl hexoside	[3]	2.71 ± 0.02
3	14.9	255,356	417	255(100)	(Iso)liquiritigenin-*O*-hexoside	[22]	1.38 ± 0.02
4	15.1	255,356	417	255(100)	(Iso)liquiritigenin-*O*-hexoside	[22]	0.71 ± 0.01
5	16.1	257,352	417	255(100)	(Iso)liquiritigenin-*O*-hexoside	[22]	0.323 ± 0.003
6	17.2	256,354	449	312(100)	Myricetin-*O*-pentoside	[1]	4.91 ± 0.08
7	17.4	350	463	317(100)	Myricetin-*O*-deoxyhexoside	[1]	2.47 ± 0.01
8	21.4	335	431	269(100)	Apigenin-*O*-hexoside	[3]	4.1 ± 0.2
9	27.5	360	719	301(100)	Ellagic acid derivative	[1]	1.56 ± 0.01
10	28.0	360	719	301(100)	Ellagic acid derivative	[1]	1.43 ± 0.01
11	30.8	362	733	301(100)	Ellagic acid derivative	-	2.17 ± 0.04
12	31.0	360	733	301(100)	Ellagic acid derivative	-	2.61 ± 0.03
Anthocyanins							
13	15.7	523	465	303(100)	Delphinidin-3-*O*-glucoside	[23]	2.98 ± 0.04
14	19.2	515	449	287(100)	Cyanindin-3-*O*-glucoside	[23]	4.68 ± 0.07
					**TPC**		**33.4 ± 0.5**
**Pulp**							
Non-anthocyanins							
2	7.0	344	325	163(100)	*p*-Coumaroyl hexoside	[3]	1.77 ± 0.01
15	8.2	326	355	193(100)	Ferulic acid hexoside	[3]	0.307 ± 0.002
16	14.3	350	479	317(100)	Myricetin-*O*-hexoside	[1]	0.613 ± 0.003
6	17.0	352	449	317(100)	Myricetin-*O*-pentoside	[1]	0.63 ± 0.01
Anthocyanins							
14	19.0	515	449	287(100)	Cyanidin-3-*O*-glucoside	[23]	1.000 ± 0.001
					**TPC**		**4.32 ± 0.03**
**Seed**							
Non-anthocyanins							
17	5.0	242,370	469	425(100),407(22),300(10)	Valoneic acid dilactone	[1]	1.893 ± 0.004
18	6.5	243,272	935	917(24),633(100),301(12)	Di-HHDP-galloyl-glucoside	[1]	1.41 ± 0.01
19	12.9	243,360	463	301(100)	Ellagic acid hexoside	[1]	2.12 ± 0.01
4	15.0	271,360	417	250(100)	(Iso)liquiritigenin-*O*-hexoside	[22]	tr
20	16.9	246,360	433	301(100)	Ellagic acid pentoside	[1]	5.59 ± 0.03
21	18.5	252,365	301	284(10),245(3),185(4),173(5),157(3),145(6)	Ellagic acid	[1]	8.57 ± 0.03
22	24.4	250,361	489	301(100)	Ellagic acetyl rhamnoside	[1]	2.07 ± 0.01
23	25.3	252,361	489	301(100)	Ellagic acetyl rhamnoside	[1]	1.75 ± 0.01
					**TPC**		**23.41 ± 0.07**

Notes: tr = traces. Phenolic compound used for quantification: apigenin-7-*O*-glucoside (y = 10683x − 45794; R^2^ = 0.996); 5-*O*-caffeoylquinic acid (y = 168823x + 161172; R^2^ = 0.999); *p*-coumaric acid (y = 301950x + 6966.7; R^2^ = 0.999); ellagic acid (y = 26719x − 317255; R^2^ = 0.999); ferulic acid (y = 633126x − 185462; R^2^ = 0.999); isoliquiritigenin (y = 42820x + 184902; R^2^ = 0.999); quercetin-3-*O*-rutinoside (y = 13343x − 76751; R^2^ = 0.999); cyanindin-3-*O*-glucoside (y = 134578x − 3000000, R^2^ = 0.999). TPC—total phenolic compounds.

**Table 2:** Antimicrobial potentials of the distinct camu-camu ethanolic extracts (peel, pulp, and seed).

	Peel		Pulp		Seed		Ampicillin (20 mg/mL)		Imipenem (1 mg/mL)		Vancomycin (1 mg/mL)		Fluconazole (1 mg/mL)	
**Antibacterial activity**	MIC	MBC	MIC	MBC	MIC	MBC	MIC	MBC	MIC	MBC	MIC	MBC	MIC	MFC
**Gram-negative bacteria**														
*Escherichia coli*	10	>20	10	>20	20	>20	<0.15	<0.15	<0.0078	<0.0078	nt	nt	nt	nt
*Klebsiella pneumoniae*	10	>20	20	>20	20	>20	10	20	<0.0078	<0.0078	nt	nt	nt	nt
*Morganella morganii*	2.5	>20	5	>20	20	>20	20	>20	<0.0078	<0.0078	nt	nt	nt	nt
*Proteus mirabilis*	10	20	10	>20	10	>20	<0.15	<0.15	<0.0078	<0.0078	nt	nt	nt	nt
*Pseudomonas aeruginosa*	5	>20	10	>20	10	>20	>20	>20	0.5	1	nt	nt	nt	nt
**Gram-positive bacteria**														
*Enterococcus faecalis*	10	>20	10	>20	20	>20	<0.15	<0.15	nt	nt	<0.0078	<0.0078	nt	nt
*Listeria monocytogenes*	5	>20	>20	>20	2.5	>20	<0.15	<0.15	<0.0078	<0.0078	nt	nt	nt	nt
MRSA	0.625	>20	10	>20	20	>20	<0.15	<0.15	nt	nt	<0.0078	<0.0078	nt	nt
MSSA	0.625	>20	10	>20	10	>20	<0.15	<0.15	nt	nt	0.25	0.5	nt	nt
**Antifungal activity**														
*Candida albicans*	20	>20	>20	>20	10	>20	nt	nt	nt	nt	nt	nt	0.06	0.06

Notes: nt, not tested. MIC, minimal inhibitory concentration; MBC, minimal bactericidal concentration; MFC, minimal fungicidal concentration; MRSA, Methicillin-resistant *Staphylococcus aureus*; MSSA, Methicillin-susceptible *Staphylococcus aureus*.

**Table 3:** Anti-proliferative and hepatotoxic potentials of the distinct camu-camu ethanolic extracts (peel, pulp, and seed).

Camu-camu	Peel	Pulp	Seed	*p*-Student *t*-Test
**Anti-proliferative activity (GI_50_, µg/mL) ^1^**				
HepG2 (hepatocellular carcinoma)	238 ± 10	297 ± 15	>400	0.001
NCI-H460 (non-small cell lung cancer)	304 ± 12	>400	>400	-
HeLa (cervical carcinoma)	180 ± 8 ^c^	196 ± 7 ^b^	320 ± 1 ^a^	-
MCF-7 (breast carcinoma)	279 ± 15	331 ± 15	>400	0.004
**Hepatotoxicity (GI_50_, µg/mL) ^1^**				
PLP2	>400	>400	>400	-

^1^ GI_50_ values correspond to the sample concentration achieving 50% of growth inhibition in human tumor cell lines or in liver primary culture PLP2. Ellipticine GI_50_ values: 1.21 µg/mL (MCF-7), 1.03 µg/mL (NCI-H460), 0.91 µg/mL (HeLa), 1.10 µg/mL (HepG2), and 2.29 (PLP2). In each row, different letters mean significant difference.

**Table 4:** Macronutrients, free sugars composition (g/100 g), and energy value (kcal/100 g) of the yogurts along shelf life.

Nutritional Parameters									
Storage Time	Sample	Moisture	Fat	Protein	Carbohydrates	Ash	Energy	Galactose	Lactose
***0 days***	Control	84.4 ± 0.1	3.78 ± 0.02	5.8 ± 0.1	5.12 ± 0.08	0.95 ± 0.03	77.6 ± 0.2	1.035 ± 0.006	2.531 ± 0.003
	Fortified	84.4 ± 0.8	3.68 ± 0.08	5.90 ± 0.09	5.1 ± 0.1	0.887 ± 0.003	77.2 ± 0.3	1.042 ± 0.009	2.57 ± 0.03
	***p*-Student *t*-Test**	0.031	0.036	0.114	0.999	0.005	0.087	0.181	0.048
***7 days***	Control	82.4 ± 0.6	4.18 ± 0.07	6.5 ± 0.1	6.0 ± 0.1	0.97 ± 0.01	87.6 ± 0.3	1.13 ± 0.04	2.8 ± 0.1
	Fortified	83.1 ± 0.3	4.02 ± 0.05	6.39 ± 0.09	5.63 ± 0.08	0.86 ± 0.03	84.3 ± 0.2	1.13 ± 0.01	2.81 ± 0.06
	***p*-Student *t*-Test**	0.001	0.011	0.145	0.002	0.001	<0.001	0.785	0.356
***15 days***	Control	81.6 ± 0.7	4.49 ± 0.03	6.8 ± 0.1	6.1 ± 0.1	1.01 ± 0.04	91.9 ± 0.2	1.22 ± 0.04	3.03 ± 0.04
	Fortified	82.8 ± 0.4	4.16 ± 0.02	6.5 ± 0.1	5.53 ± 0.02	1.02 ± 0.04	85.68 ± 0.05	1.15 ± 0.04	2.850 ± 0.005
	***p*-Student *t*-Test**	<0.001	<0.001	0.019	0.001	0.702	<0.001	0.034	<0.001

In each column and within each storage time a Student’s *t*-test was used to determine the significant difference between two different samples, with α = 0.05: *p* < 0.001 means a significant difference between the sample.

**Table 5:** Fatty acids composition of the yogurts, in relative percentage of each fatty acid, along shelf life.

	Storage Time								
	*0 days*			*7 days*			*15 days*		
	Control	Fortified	*p*-Student´s *t*-Test	Control	Fortified	*p*-Student´s *t*-Test	Control	Fortified	*p*-Student´s *t*-Test
**C4:0**	5.02 ± .02	6.29 ± 0.01	<0.0001	4.186 ± 0.003	3.359 ± 0.008	<0.001	3.39 ± 0.07	3.293 ± 0.001	0.034
**C6:0**	3.379 ± 0.001	4.81 ± 0.07	<0.0001	3.10 ± 0.02	3.14 ± 0.02	0.034	3.138 ± 0.001	2.960 ± 0.005	<0.001
**C8:0**	1.78 ± 0.01	2.41 ± 0.02	<0.0001	1.596 ± 0.004	1.709 ± 0.008	<0.001	1.68 ± 0.02	1.628 ± 0.001	0.002
**C10:0**	3.465 ± 0.008	4.33 ± 0.01	<0.0001	3.215 ± 0.001	3.238 ± 0.006	0.001	3.427 ± 0.014	3.34 ± 0.01	<0.001
**C11:0**	0.094 ± 0.008	0.099 ± 0.009	0.367	0.098 ± 0.001	0.077 ± 0.001	<0.001	0.082 ± 0.007	0.084 ± 0.006	0.617
**C12:0**	3.635 ± 0.04	4.10 ± 0.03	<0.0001	3.534 ± 0.001	3.412 ± 0.008	<0.001	3.74 ± 0.01	3.698 ± 0.003	0.001
**C13:0**	0.103 ± 0.001	0.111 ± 0.001	<0.0001	0.113 ± 0.004	0.087 ± 0.003	<0.001	0.102 ± 0.001	0.105 ± 0.001	0.007
**C14:0**	11.42 ± 0.05	11.217 ± 0.01	<0.0001	11.42 ± 0.02	11.00 ± 0.03	<0.001	11.87 ± 0.02	11.97 ± 0.01	<0.001
**C14:1**	1.17 ± 0.01	1.145 ± 0.008	0.013	1.17 ± 0.01	1.09 ± 0.03	0.003	1.20 ± 0.03	1.20 ± 0.01	0.877
**C15:0**	1.286 ± 0.007	1.20 ± 0.02	0.001	1.292 ± 0.009	1.279 ± 0.006	0.047	1.323 ± 0.008	1.351 ± 0.001	0.001
**C15:1**	0.271 ± 0.008	0.25 ± 0.01	0.004	0.263 ± 0.001	0.263 ± 0.005	0.824	0.272 ± 0.008	0.275 ± 0.001	0.435
**C16:0**	32.21 ± 0.06	30.35 ± 0.07	<0.0001	33.239 ± 0.004	33.22 ± 0.06	0.452	33.33 ± 0.06	33.778 ± 0.008	<0.001
**C16:1**	1.365 ± 0.004	1.27 ± 0.01	<0.0001	1.391 ± 0.001	1.352 ± 0.006	<0.001	1.370 ± 0.005	1.393 ± 0.009	0.006
**C17:0**	0.608 ± 0.002	0.57 ± 0.02	0.006	0.630 ± 0.005	0.641 ± 0.004	0.011	0.624 ± 0.002	0.629 ± 0.001	0.006
**C17:1**	0.243 ± 0.001	0.22 ± 0.01	0.005	0.246 ± 0.001	0.254 ± 0.001	<0.001	0.241 ± 0.006	0.25 ± 0.002	0.026
**C18:0**	9.66 ± 0.02	9.726 ± 0.007	0.002	10.132 ± 0.003	10.473 ± 0.001	<0.001	9.824 ± 0.006	9.872 ± 0.006	<0.001
**C18:1n9**	19.72 ± 0.02	17.2 ± 0.2	<0.0001	20.12 ± 0.02	20.64 ± 0.03	<0.001	19.60 ± 0.05	19.737 ± 0.004	0.002
**C18:2n6**	2.13 ± 0.05	2.13 ± 0.03	0.844	2.083 ± 0.001	2.33 ± 0.08	0.002	2.28 ± 0.05	2.21 ± 0.02	0.029
**C18:3n3**	1.672 ± 0.001	1.57 ± 0.02	<0.0001	1.460 ± 0.001	1.654 ± 0.004	<0.001	1.59 ± 0.01	1.578 ± 0.009	0.226
**C20:0**	0.133 ± 0.004	0.145 ± 0.001	0.003	0.17 ± 0.02	0.126 ± 0.006	0.004	0.142 ± 0.001	0.149 ± 0.001	<0.001
**C20:1**	0.04 ± 0.01	0.043 ± 0.001	0.016	0.039 ± 0.001	0.041 ± 0.001	0.001	0.039 ± 0.001	0.039 ± 0.001	0.999
**C20:3n6**	0.16 ± 0.01	0.199 ± 0.006	0.003	0.236 ± 0.003	0.13 ± 0.01	<0.001	0.314 ± 0.001	0.14 ± 0.01	<0.001
**C20:4n6**	0.200 ± 0.001	0.32 ± 0.02	<0.0001	0.171 ± 0.001	0.24 ± 0.02	0.001	0.26 ± 0.01	0.174 ± 0.004	<0.001
**C20:3n3**	0.16 ± 0.01	0.138 ± 0.008	0.028	0.006 ± 0.001	0.13 ± 0.02	<0.001	0.077 ± 0.002	0.058 ± 0.006	0.002
**C22:0**	0.084 ± 0.01	0.128 ± 0.001	<0.0001	0.112 ± 0.002	0.11 ± 0.02	0.852	0.106 ± 0.003	0.099 ± 0.001	0.003
**SFA**	72.87 ± 0.07	75.5 ± 0.2	<0.0001	72.82 ± 0.01	71.87 ± 0.04	<0.001	72.76 ± 0.01	72.96 ± 0.04	<0.001
**MUFA**	22.812 ± 0.003	20.2 ± 0.2	<0.0001	23.220 ± 0.007	23.65 ± 0.04	<0.001	22.72 ± 0.06	22.895 ± 0.006	0.003
**PUFA**	4.32 ± 0.07	4.36 ± 0.03	0.274	3.955 ± 0.004	4.49 ± 0.08	<0.001	4.51 ± 0.05	4.15 ± 0.03	<0.001

The results are presented as mean ± SD. Butyric acid (C4:0); Caproic acid (C6:0); Caprylic acid (C8:0); Capric acid (C10:0); Undecylic acid (C11:0); Lauric acid (C12:0); Tridecylic acid (C13:0); Myristic acid (C14:0); Myristoleic Acid (C14:1); Pentadecanoic acid (C15:0); cis-10-Pentadecenoic acid (C15:1); Palmitic acid (C16:0); Palmitoleic acid (C16:1); Heptadecanoic acid (C17:0); cis-10-Heptadecenoic acid (C17:1); Stearic acid (C18:0); Oleic acid (C18:1n9); Linoleic acid (C18:2n6); α-Linolenic acid (C18:3n3); Arachidic acid (C20:0); cis-11-Eicosenoic acid (C20:1); homo-γ-Linolenic acid (C20:3n6); Arachidonic acid (C20:4n6); cis-11, 14, 17 Eicosatrienoic acid (C20:3n3); Behenic acid (C22:0); SFA-Saturated fatty acids; MUFA-Monounsaturated fatty acids; PUFA-Polyunsaturated fatty acids. In each row and within each storage time a Student´s *t*-test was used to determine the significant difference between two different samples, with α = 0.05: *p* < 0.001 means a significant difference between the samples.
